# Physico-Mechanical Properties and Bonding Performance of Graphene-Added Orthodontic Adhesives

**DOI:** 10.3390/jfb15080204

**Published:** 2024-07-24

**Authors:** Shiyao Liu, Ahmed El-Angbawi, Vinicius Rosa, Nick Silikas

**Affiliations:** 1 Division of Dentistry, University of Manchester, Manchester M13 9PL, UK; shiyao.liu@manchester.ac.uk (S.L.); ahmed.el-angbawi@manchester.ac.uk (A.E.-A.); 2 Centre for Advanced 2D Materials and Graphene Research Centre, National University of Singapore, Singapore 119085, Singapore; vini@nus.edu.sg; 3 Faculty of Dentistry, National University of Singapore, 11 Lower Kent Ridge Road, Singapore 119085, Singapore; 4 Oral Care Health Innovations and Design Singapore (ORCHIDS), National University of Singapore, 11 Lower Kent Ridge Road, Singapore 119085, Singapore

**Keywords:** graphene, orthodontic adhesives, retainer, bonding strength, stickiness, degree of conversion

## Abstract

This study aimed to assess the key physico-mechanical properties and bonding performance of orthodontic adhesives with graphene addition for bonding a fixed retainer. Transbond LR (3M) and Transbond LV (3M) with no graphene were set as the control groups. Graphene was added into LR and LV at concentrations of 0.01 wt%, 0.05 wt% and 0.1 wt%. The stickiness of the uncured samples (*n* = 5) and real-time degree of conversion (*DC*) of the samples (*n* = 3) were measured over a 24-h period using Fourier-transform infrared spectroscopy. The hardness and other mechanical parameters, including the Martens hardness (*HM*), indentation modulus (*E_IT_*), elastic index (*η_IT_*) and creep (*C_IT_*), were measured (*n* = 5). To measure the shear bond strength (SBS), adhesive composites were applied using a mold to bond the retainer wire to the lingual surfaces of bovine incisors (*n* = 10). Fracture modes subsequent to the SBS test were examined under light microscopy. Statistical analysis was conducted using ANOVA and Tukey tests (α = 0.05). In the LR groups, the LR + 0.01 showed the highest SBS (12.6 ± 2.0 MPa) and *HM* (539.4 ± 17.9 N/mm^2^), while the LV + 0.05 (7.7 ± 1.1 MPa) had the highest SBS and the LV + 0.1 had the highest *HM* (312.4 ± 17.8 N/mm^2^) among the LV groups. The most frequent failure mode observed was adhesive fracture followed by mixed fracture. No statistical difference was found between the graphene-added groups and the control groups in terms of the *E_IT_*, *η_IT_* and *C_IT_*, except that the *C_IT_* was significantly lower in the LR + 0.01 than in the control group. Graphene addition had no significant adverse effect on the stickiness and *DC* of both LR and LV.

## 1. Introduction

Orthodontic treatment realigns teeth to improve alignment and occlusion. However, after treatment, the surrounding tissues need time to adapt. During this period, there is a risk of unwanted tooth movement. Therefore, retention is crucial to prevent post-treatment dental changes. Wearing fixed retainers is a preferred retention method because it requires less patient compliance [1]. Fixed retainers are bonded to the lingual tooth surface with resin composite orthodontic adhesives. Currently, many clinicians prefer to employ fixed retainers for long periods of time, sometimes even for the lifetime of patients [2,3]. To ensure the long-term durability of fixed retainers, it is essential to improve the physico-mechanical properties of orthodontic adhesives.

Retainer detachment has been identified as the primary cause of retainer failure [4]. The detachment happens at the interface between the enamel surface, composite and wire. According to a recent systematic review, detachment at the interface between the adhesive composite and enamel is most common [5]. Therefore, the adhesive should possess sufficient resistance to endure the forces of mastication and the deformation caused by the stretching of orthodontic wires.

In addition to good mechanical properties, orthodontic adhesives are also expected to be easy to use clinically. Flowable orthodontic adhesives have subsequently been introduced, allowing for precise application facilitated by dispensing the adhesive through a needle tip attached to a syringe [6]. Flowable composites come in various formulations and viscosities. Typically, they are not sticky and can eliminate the need for trimming and polishing to reduce chair time [7,8,9]. Moreover, they are expected to better permeate into the enamel, resulting in an improved bonding interface. The increased flowability is primarily achieved by reducing the filler content [10,11]. However, this decrease in the filler content could compromise the mechanical performance of the materials [11,12,13].

It is well established that by incorporating inorganic fillers into composite polymers, they will enhance their mechanical properties, including the hardness, bonding strength and flexural strength [14]. Recently, great interest has been shown in the application of graphene as the reinforcing phase of dental resin materials [15,16]. Due to its exceptional strength and stiffness, graphene reinforces the resin matrix to increase the overall mechanical properties [17]. The nanoscale of graphene also allows reinforcement on a molecular level [18,19,20]. It has been suggested that a small amount of graphene addition can lead to a significant improvement in composites’ properties, especially the mechanical properties [21,22,23,24]. Another advantage is the relatively low economic cost of producing graphene, which makes it more attractive for clinical applications.

The aim of this study was to investigate the effect of increasing amounts of graphene on the key physico-mechanical properties and the retainer bonding performance of orthodontic adhesives (Transbond LR and Transbond LV). The null hypotheses were that (1) different graphene additions would not significantly affect the degree of conversion (*DC*), stickiness, hardness and relevant parameters of the materials; (2) regardless of the material, the shear bond strength (SBS) would not change with different graphene additions.

## 2. Materials and Methods

### 2.1. Preparation of the Modified Experimental Adhesives with Graphene

Two commonly used commercial orthodontic composite adhesives were used as follows: Transbond™ LR Light Cure Adhesive (LR, 3M-Unitek™, St. Paul, MN, USA), a specific retainer adhesive, and Transbond™ Supreme LV Low Viscosity Light Cure Adhesive (LV, 3M-Unitek™, St. Paul, MN, USA), a flowable orthodontic adhesive. The graphene was supplied by the Center for Advanced 2D Materials at the National University of Singapore and has been characterized in a previous study [25]. Graphene powder was weighed utilizing an electronic balance (Ohaus Analytical Plus, Ohaus, Parsippany, NJ, USA) to a precision of 0.01 mg. Graphene was mixed with LR and LV at concentrations of 0.01, 0.05 and 0.1 wt% in a speedmixer (DAC 150.1 FVZK, High Wycombe, Buckinghamshire, UK) at 3500 rpm for 5 min. LR and LV with no graphene were the control groups. The prepared adhesives were used within one month.

### 2.2. Stickiness

**Figure 1:**
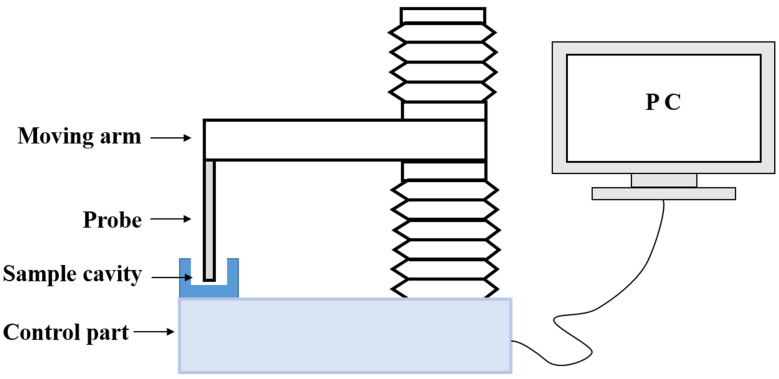
Schematic diagram of the texture analyzer used to measure the stickiness.

**Figure 2:**
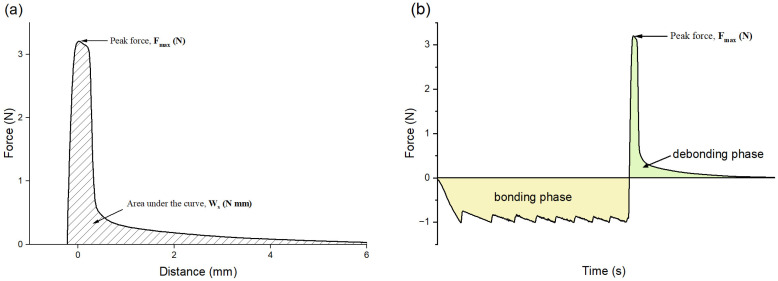
Representative diagrams showing the measurement of stickiness. (**a**) Force–distance curve, (**b**) force–time curve.

The stickiness of the composites was measured using a Texture Analyzer (TA.XT2i, Stable Micro Systems, Godalming, Surrey, UK). Composites from each group were added into a cavity (*n* = 5). A cylindrical stainless-steel probe (φ = 6 mm) with a flat end was immersed into the composites at a descent rate of 0.5 mm/s, and data acquisition began once the probe contacted the composites (Figure 1). The probe continued to descend until a compressive force of 1 N was achieved, which was then held constant for 1 s. Subsequently, the probe was elevated vertically at 2 mm/s to simulate the removal of dental instruments. During this process, the composites adhered to the probe and experienced a tensile force until they reached the interfacial strength, causing the composite paste to separate from the probe. Stickiness refers to the maximum separation force *F_max_* (N) and the work of probe separation *W_s_* (N mm) required when the probe moves backward (Figure 2) [26,27,28].

### 2.3. Measurement of Real-Time Degree of Conversion

The *DC* (*n* = 3) was measured using a Fourier-transform infrared (FTIR) spectrometer (ALPHA II, Bruker, Billerica, MA, USA) with a single reflection ATR accessory. Spectra were collected under the following conditions: 4 cm^−1^ resolution, 4000–400 cm^−1^ wavenumber range, one spectrum every 5 s. A cylindrical mold with 4 mm diameter and 1 mm thickness was positioned over the ATR crystal. Uncured composite pastes were placed into the mold and pressed from the top with a Mylar strip and a glass slide to eliminate air bubbles and remove extra materials. After removing the glass slide, spectral acquisition was initiated, and the initial spectra of the uncured composites were recorded. The samples were then immediately cured with a light-curing unit (LCU, Elipar™ S10, 3 M ESPE, St. Paul, MN, USA) at an output of 1200 mW/cm^2^ for 20 s. Real-time spectra were continuously collected for 24 h using OPUS software (version 8.1, BRUKER OPTIK GmbH, Ettlingen, Germany) while the samples remained in position on the ATR crystal [29,30]. To calculate the *DC*, the absorption peak area of the aliphatic C=C at 1637 cm^−1^ as the analytical frequency and the aromatic C=C absorbance at 1608 cm^−1^ as the internal reference were utilized according to the following equation:

$$DC\%=\left[1-\frac{{\left(\frac{aliphatic\;C=C}{aromatic\;C=C}\right)}_{polymerized}}{{\left(\frac{aliphatic\;C=C}{aromatic\;C=C}\right)}_{unpolymerized}}\right]\times 100\%$$

### 2.4. Instrumented Indentation Test (IIT)

The IIT has been developed to test the mechanical properties of materials by consistently monitoring the force and depth during the loading cycle [31]. Compared with the conventional hardness testing method, the IIT provides more information on the elastic–viscoplastic response of the material [32]. Many material parameters other than the hardness can be calculated. The specimens for the IIT measurements were fabricated using a cylindrical mold with 15 mm diameter and 1 mm height (*n* = 5). The same LCU (Elipar™ S10) was used to cure. Each sample was wet-ground with increasing grit sizes of p1000, p1200, p2500, p4000 silicon carbide papers. Specimens were tested 1 h after curing. The instrumented indentation test at the macro scale was carried out with a universal hardness tester (ZHU 2.5, ZwickRoell Ltd., Leominster, UK). Five force–displacement curves were recorded from each specimen using a Vickers indenter with a 10 N load and a 15 s dwell time. The loading and unloading rate were set as 5 N/s. The parameters were calculated by testXpert according to equations presented in ISO 14577 [33].

Martens hardness (*HM*) was determined by dividing the test force by the surface area of the indentation created under the applied test force:

$$HM=\frac{F}{A_{S}(h)}=\frac{F}{26.43\times h^{2}}$$

where *HM* (Martens hardness) is in N/mm^2^, *F* (test force) is in N, $A_{S}(h)$ (surface area of the indenter at distance *h* from the tip) is in mm^2^.

The indentation modulus (*E_IT_*) was calculated using the following equation:

$$E_{IT}=\left(1-\nu_{S}^{2}\right)/\left(\frac{1}{E_{r}}-\frac{\left(1-\nu_{i}^{2}\right)}{E_{i}}\right)$$

where $E_{IT}$ (indentation modulus) is in KN/mm^2^, $E_{r}$ (reduced modulus of the indentation contact) is in KN/mm^2^, $E_{i}$ (modulus of the indenter) is 1.14 × 10^3^ KN/mm^2^ for diamond, ν_*s*_ (Poisson’s ratio of the test piece) and *ν_i_* (Poisson ratio of indenter) with *ν_s_* = 0.3 and *ν_i_* = 0.07.

The elastic index ($\eta_{IT}$), the ratio of elastic to total indentation work, can be quantified by computing the areas under the loading and unloading curves:

$$\eta_{IT}=\frac{W_{elastic}}{W_{total}}\times 100$$

where $\eta_{IT}\;$(elastic index) is in %, *W_elastic_* (elastic resilience work) is in N·m, *W_total_* (total indentation work) is in N·m.

The creep (*C_IT_*) was defined as the percentage increase in the indentation depth under constant loading over a given period of time and calculated by the following equation:

$$C_{IT}=\frac{h_{2}-h_{1}}{h_{1}}\times 100\%$$

where *h*_1_ (indentation depth after having reached the test load) is in mm and *h*_2_ (indentation depth after the expiry of the waiting time) is in mm.

### 2.5. Shear Bond Strength

Eighty freshly extracted bovine incisors (*n* = 10) were selected in this study. Teeth exhibiting cracks, hypoplastic enamel, caries or those pretreated with chemical agents were excluded. Additionally, soft-tissue remnants and calculus were removed. All procedures were carried out by the same operator. The root of teeth was embedded into a mold using chemically cured acrylic resin. All teeth were stored in deionized water under 5 °C and used within three months.

The enamel surface was etched with 37% phosphoric acid for 30 s, rinsed for 20 s and dried until a frosty white appearance of the etched enamel was observed. The specimens were then divided into eight groups randomly. To standardize the amount of adhesive resin applied each time, a dome-shape mini-mold wire bonder (diameter, 3 mm; DB Orthodontics Ltd., Silsden, UK) was used to place the adhesive on the teeth. An 8 mm long two single-strand wire (Ortho-Care Ltd., Bradford, West Yorkshire, UK) was located in the middle of the adhesive resin. Then, the resin was cured for 20 s using the same LCU. 

The SBS was measured by a universal testing machine with a 500 N load cell (Zwick/Roell Z020, Leominster, UK). The samples were secured in the jig attached to the machine, and load was then applied to each specimen at the interface between the tooth and composites by the blade at a crosshead speed of 1 mm/min until the debonding. To determine the bond strength in MPa, the maximum load (N) was divided by the cross-sectional area of the bonded composite.

### 2.6. Fracture Analysis

After the SBS test, the tooth surface was examined at ×10 magnification with a microscope (Revolve, Echo, A BICO Co., San Diego, CA, USA) to determine the fracture mode. The fracture mode was classified as follows: adhesive failure, fracture occurring between composite resin and enamel; cohesive resin failure, fracture occurring between retainer and adhesive resin, partial adhesive remains on enamel; cohesive enamel failure, fracture occurring on the tooth with partial removal of enamel; mixed failure, more than one type of fracture exhibits in certain areas. Representative scanning electron microscope (SEM) images were obtained (Quanta 250, FEI Company, Eindhoven, The Netherlands). Samples were coated with platinum (Sputter Coater 108auto, Cressington, UK) and dried for 24 h. Images were obtained in secondary electron mode at 300 × and 1000 × magnification.

### 2.7. Statistical Analysis

All data were analyzed with SPSS (version 25.0, SPSS Inc., Chicago, IL, USA). The data for *DC*, *F_max_*, *W_s_*, *HM*, *E_IT_*, *η_IT_*, *C_IT_* and SBS between different graphene concentration groups were statistically analyzed with one-way analyses of variance (ANOVA) and Tukey’s post hoc test (α  =  0.05). The Student’s *t*-test was used to compare the significant differences between materials (α  =  0.05).

## 3. Results

### 3.1. Stickiness

**Figure 3:**
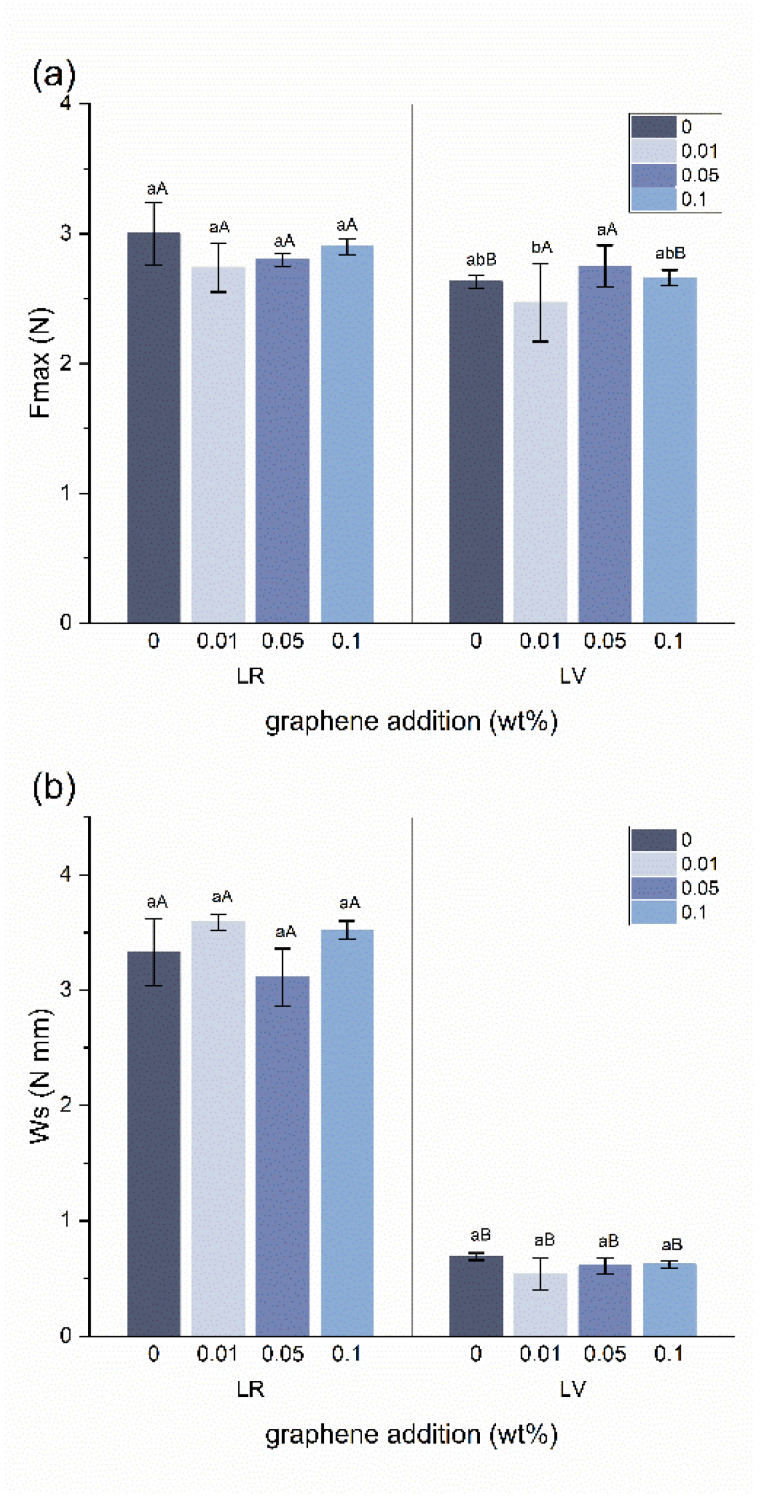
Differences in (**a**). *F_max_* and (**b**). *W_s_*. Within each material, significant differences between graphene concentrations are indicated by different lower-case letters. Within each graphene concentration, significant differences between materials are indicated by different upper-case letters.

Figure 3 shows the stickiness (*F_max_* and *W_s_*) for LR and LV with different graphene concentrations. There was no statistically significant difference in *F_max_* and *W_s_* between materials with different graphene concentrations and their control group in either the LR or LV groups. *F_max_* was consistently lower in the LV groups than in the LR groups, but the difference was statistically significant only in the 0.1 wt% graphene-added group. The LR groups have a significantly higher *W_s_* value compared with the LV group (*p* < 0.05).

### 3.2. Real-Time Degree of Conversion

**Figure 4:**
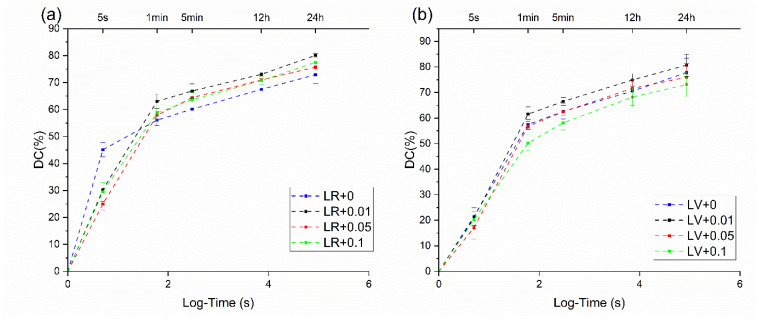
*DC* (%) as a function of log-time up to 24 h for (**a**) LR, (**b**) LV.

Figure 4 shows the real-time *DC* in 24 h of the samples. LR with no graphene addition showed a higher *DC* 5s after light initiation. However, there was no difference between the graphene-added group and the control group after 1 min. For both materials, all groups showed a similar trend regarding the change in the real-time *DC*. 

### 3.3. Martens Hardness and Parameters

**Figure 5:**
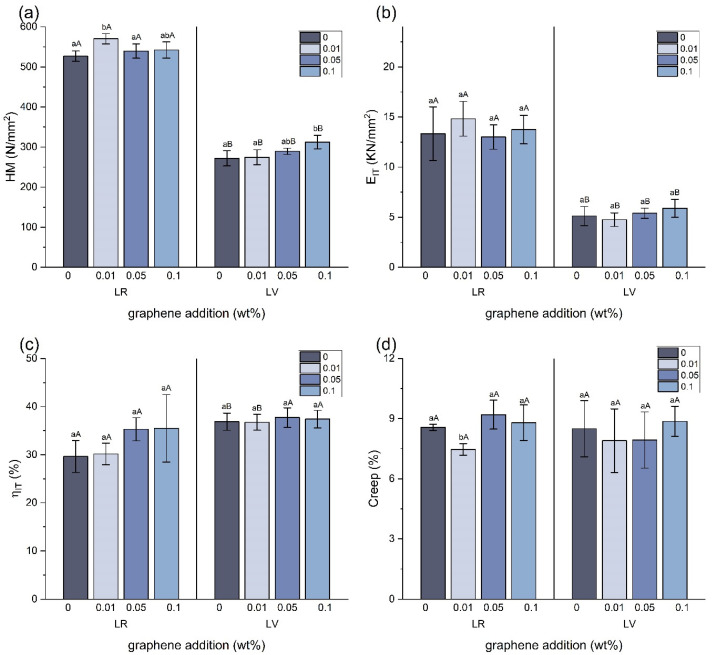
Mean and standard deviation values of (**a**). Martens hardness (*HM*), (**b**). indentation modulus (*E_IT_*), (**c**). elastic index (*η_IT_*), (**d**). creep (*C_IT_*). Within each material, significant differences between graphene concentrations are indicated by different lower-case letters. Within each graphene concentration, significant differences between materials are indicated by different upper-case letters.

The results of the *HM* and parameters are presented in Figure 5. The LR + 0.01 group exhibited the highest hardness, showing an 8.3% and 11.2% increase in the hardness and indentation modulus compared with LR. In the LV groups, the LV + 0.1 was the hardest, with a remarkable 14.9% increase in the hardness and a 15.0% increase in the indentation modulus compared to the control group. However, in the LR groups, the hardness and modulus decreased with the addition of higher concentrations of graphene, although there were no significant differences with the control group. In both the LR and LV groups, no significant differences in *η_IT_* were identified between the control group and graphene-added groups. The creep values ranged from 7.47 to 9.2% for the LR groups and 7.9 to 8.87% for the LV groups. A significant difference was only observed in the LR + 0.01 (7.47%) compared with the other LR groups.

### 3.4. Shear Bond Strength

**Figure 6:**
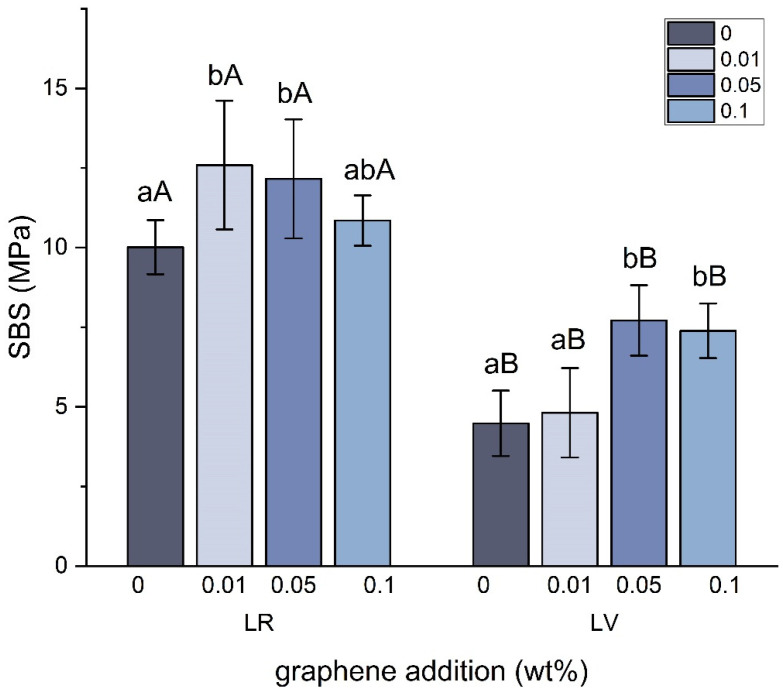
Mean and standard deviation values of shear bond strength (SBS). Within each material, significant differences between graphene concentrations are indicated by different lower-case letters. Within each graphene concentration, significant differences between materials are indicated by different upper-case letters.

From Figure 6, it can be seen that the addition of graphene increased the shear bond strength of both LR and LV. In the LR groups, a statistically significant enhancement in the SBS was found after adding 0.01 and 0.05 wt% graphene. The groups with 0.1 wt% graphene also showed an increased SBS, but not statistically significant. In the LV groups, the SBS increased with the increasing graphene addition. Specifically, the LV + 0.05 and LV + 0.1 groups had a significantly higher SBS compared to the control. Regardless of the graphene concentration, the SBS was consistently higher in the LR group than in the LV group.

### 3.5. Fracture Analysis

**Figure 7:**
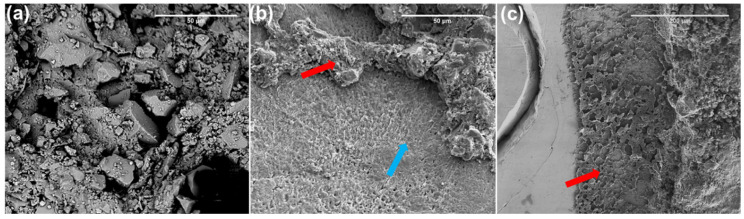
Representative SEM images after SBS test. (**a**). Fractured orthodontic adhesive after cohesive resin failure, (**b**) mixed failure, blue arrow showing the enamel surface and the red arrow showing the remained orthodontic adhesive, (**c**) surface of retainer, retainer, red arrow showing the remained orthodontic adhesive.

The spreading of the fracture mode is presented in Table 1. Typical SEM images after fracture are shown in Figure 7. Adhesive failure was the most common failure type in all groups, followed by mixed failure. None of the specimens showed cohesive enamel failure. The most adhesive failures occurred with the control groups.

### 3.6. Discussion

In this study, graphene was added to two widely used commercial orthodontic adhesives, LR and LV. The results have shown that the incorporation of graphene in orthodontic adhesives did not adversely affect the stickiness and *DC*, while improving the hardness and shear bond strength at certain concentrations. Therefore, both null hypotheses are rejected.

The properties were chosen in order to simulate the clinical pathway of these materials. It started with the pre-setting, handling properties (stickiness). Then, immediately after placement and light curing, the short-term properties (*DC*, *HM* and other mechanical parameters, SBS and fracture analysis). These can provide a more holistic approach in trying to predict the clinical behavior. 

Stickiness is an essential clinical concept that characterizes the adhesion of resin composites to dental placement instruments. It is desirable for materials to be easily handled, allowing them to flow onto the tooth surface and remain in place after the removal of dental instruments [34,35]. This minimizes the risk of voids caused by instrument pull-back procedures. However, those flowable composites tend to have a reduced mechanical performance compared with packable composites with a higher filler loading [36,37]. There have been studies on the addition of nanoparticles to resins to improve their mechanical properties [38,39,40], but the stickiness was inevitably affected by changing the filler loading [34,41,42]. Reassuringly, the addition of graphene had no significant effect on the stickiness of the two orthodontic adhesives at any concentration compared with the control groups in this study (Figure 3). This could be attributed to the relatively small amount of graphene added. Additionally, the inherent properties of the adhesives, such as their viscosity and intermolecular force, likely dominated the stickiness behavior, overshadowing the minor influence of the relatively small amounts of graphene added. In this study, a texture analyzer was used to measure the stickiness and was represented by two parameters, *F_max_* and *W_s_*. This method has been proved with good reproducibility [41,42]. 

Degree of conversion (*DC*) refers to the percentage of monomer molecules in a resin composite that have been converted into polymer chains during the curing process. It is a crucial measure for dental resin composites, as it correlates with higher mechanical properties, better long-term performance and enhanced biocompatibility. At the beginning of the light curing, the *DC* usually rises rapidly and gradually stabilizes [29,43]. Real-time degree of conversion can provide comprehensive information on the changes in the DC at the initial and completed stage of curing. The result of the real-time *DC* (Figure 4) showed that the group without graphene reached a higher *DC* at the beginning of the light exposure. A longer initial curing time may be necessary to ensure sufficient early bond strength for graphene-added adhesives. Clinicians should be mindful of the handling and placement of orthodontic appliances immediately after curing to avoid early debonding. A previous study has also shown that the addition of graphene resulted in a significant decrease in light transmission [44]. However, after 24 h, the *DC* of the graphene-added groups was comparable or even higher than that of the control groups. A high *DC* is usually considered important to ensure the mechanical properties, biocompatibility and durability of the resin composites. Although not many studies have been conducted on the effect of the addition of graphene family materials on the *DC* of dental resins, there have been some conflicting results and opinions. Sarosi et al. [45] found that the addition of graphene–gold particles increased the *DC* of dental composites and explained the result by the high thermal conductivity of the material, which led to better polymerization. Another study by Almutairi et al. [46] showed that the incorporation of graphene oxide/calcium phosphate nanofiller significantly decreased the *DC* of a dentin adhesives. It was suggested that the addition of nano fillers reduced the space for light transmission. A possible explanation for the increasing *DC* in the graphene addition groups is that graphene edges have been found to chemically bond to monomers during polymerization in the presence of free radical molecules, therefore improving the *DC* [47]. Many factors affect the results of *DC* alteration by graphene, such as the incorporation of other particles, the number of layers of graphene and the dispersion of graphene in resin [17,48]. Future research can be conducted to understand the complex mechanisms of the effects of graphene on the *DC*.

The addition of 0.01 wt% graphene in LR and 0.1 wt% in LV significantly increased the hardness of two commercial orthodontic adhesives. This corroborates previous studies suggesting that graphene can improve the mechanical properties of dental resin materials [49,50]. Graphene has a large amount of surface area on a microscopic scale, which allows it to better interact with polymers. This increased surface area helps to improve the surface hardness of the composites. However, the hardness did not increase with the increasing graphene addition in LR, which is similar to other studies involving graphene family materials [51,52,53,54]. This could be attributed to the presence of graphene agglomerates and voids in the composites. For LV, its flowability could potentially facilitate a more uniform mixture with graphene. The indentation modulus showed a similar trend to the hardness. In the context of material science, the 11.2% (LR + 0.01 group) and 15% (LV + 0.1 group) increase in the indentation modulus can be considered significant. Clinically, retainer adhesives with a high hardness and indentation modulus are less prone to wear and deformation over time, facilitating efficient force transfer from the retainer to the teeth. This extends the lifespan of the retainer and helps control relapse. Meanwhile, the ability of graphene particles to impede crack progression correlates with the enhancement in the SBS. However, it is important to note that the increase was not statistically significant within the scope of the experimental setup in this study. Compared with the conventional hardness measurement, the IIT gives information on the elastic and plastic deformation of materials [55]. In resin composites, it is suggested that as the filler content increases, the creep decreases [56]. The variations in the creep appeared to be the opposite of the hardness. The groups incorporating 0.01 wt% graphene exhibited the lowest indentation creep, indicating reduced deformation over time and enhanced dimensional stability.

The bonding strength between orthodontic adhesives and enamel is determined by both the adhesive strength and cohesive strength. There are three main adhesive mechanisms controlling the adhesion between adhesives and enamel: mechanical interlocking, where adhesive resin interlocks with the enamel microscopic porosities; adsorption, where secondary bonding forces lead to the proximity of the two phases; and chemical bonding, characterized by the formation of bonds between functional monomers and hydroxyapatite [57,58]. Cohesive strength is the internal bonding force between particles within a material. A high cohesive strength helps maintain the bond integrity under loads [57]. In this study, the SBS was significantly improved in the LR groups with 0.01 wt% (12.6 ± 2.0 MPa) and 0.05 wt% (12.2 ± 1.9 MPa) graphene, and LV with 0.05 wt% (7.7 ± 1.1 MPa) and 0.1 wt% (7.4 ± 0.9 MPa) graphene. The reason for this improvement could be similar with graphene oxide and other nanoparticles, where weak intermolecular forces and mechanical interlocking sites might be increased by the introduction of graphene [59,60]. In addition, graphene can act as a reinforcement phase to increase the cohesion of the material and impede crack propagation [61]. However, the SBS did not continue to increase in both the LR and LV groups due to the agglomeration of graphene and the increase in opacity. Additionally, the trends of the SBS and hardness did not precisely align. For example, the LV + 0.1 had a lower SBS than that of the LV + 0.05, despite displaying a higher hardness. This indicates that variations in the bonding strength are influenced by factors beyond only the material hardness [62]. Previous studies have investigated the relation between adhesive hardness and bonding strength. Kusakabe et al. [63] found a moderate correlation between the thin-film bond strength and the indentation hardness of the bonding agents. Another study conducted by Takahashi et al. [64] showed that the bond strength and the mechanical properties of the material were not correlated. As previously mentioned, the addition of graphene as a reinforcement phase enhances the hardness and indentation modulus, thereby improving the cohesive strength; however, its impact on the adhesion strength remains unclear. 

In the literature, the SBSs of these two adhesives were in the range of 6.78 [65]–24.77 [66] MPa and 4.24 [67]–15.38 [68] MPa. The wide range is mainly because the SBS results can be influenced by various factors, such as the type of primer, retainer and aging period [69,70,71]. In this study, the SBS values of LR (10.0 ± 0.9 MPa) and LV (4.5 ± 1.0 MPa) appear relatively low compared with the data from other studies. This may be due to no primer being applied in this study, as well as a hemispherical rather than the usual cylindrical mold being used to simulate the clinical conditions. Additionally, bovine teeth were used in this study, which may differ from the results of experiments using human teeth [72]. 

From the results of the failure modes observed under microscopy, adhesive fracture was the most common type of failure in all groups, where the material bonds stronger to the metal wire and may not bond sufficiently to the enamel. Less adhesive failure was observed in the graphene addition groups, indicating the increase in the bonding strength. Generally, the detaching force in adhesive failure represents the actual bonding strength between the enamel and composite [73]. Additionally, mixed failure is relatively common. Cohesive resin was a rare type of failure, indicating the materials presented high molecular forces, and the force at adhesion failure did not reach a strength that was sufficient to break the cohesion force within the material. No cohesive enamel failure was found, indicating that the bonding strength did not exceed the cohesive strength of the enamel.

There are a few limitations to this study. Firstly, the study primarily focused on short-term properties. Long-term studies are necessary to evaluate the performance of the adhesives over extended periods. Secondly, exploring a wider range of graphene concentrations could provide more comprehensive insights into its behavior in dental resin composites. Additionally, graphene may behave differently in various resin composites, and more studies should be conducted to evaluate the enhancement in graphene in dental resins. Nevertheless, graphene has the potential to be a reinforcement particle for orthodontic adhesives.

## 4. Conclusions

In conclusion, this study presents first-hand knowledge of graphene application in orthodontic retainer adhesives. The addition of graphene at various concentrations was found to enhance the short-term hardness, indentation modulus and shear bond strength of the two tested orthodontic adhesives without adversely affecting their stickiness. However, it was observed that the mechanical properties did not continuously improve with the increasing graphene concentration. Future research could optimize the exfoliation and mixing techniques of graphene to minimize the adverse effect of agglomeration on the properties. Additionally, the real-time degree of conversion measurements revealed that the addition of graphene leads to a decrease in the degree of polymerization of the composites at the initial stage of light curing, suggesting that sufficient irradiation should be ensured at the curing stage. The present study introduces a new strategy for developing orthodontic retainer adhesives.

## Figures and Tables

**Table 1:** Frequency distribution of fracture mode in all groups obtained after SBS test.

	Adhesive	Cohesive Resin	Cohesive Enamel	Mixed
LR + 0	7	1	0	2
LR + 0.01	5	0	0	5
LR + 0.05	5	0	0	5
LR + 0.1	4	2	0	4
LV + 0	6	1	0	3
LV + 0.01	6	0	0	4
LV + 0.05	5	1	0	4
LV + 0.1	5	2	0	3

## Data Availability

The original contributions presented in this study are included in the article; further inquiries can be directed to the corresponding authors.
